# Dominant bacterial phyla in caves and their predicted functional roles in C and N cycle

**DOI:** 10.1186/s12866-017-1002-x

**Published:** 2017-04-11

**Authors:** Surajit De Mandal, Raghunath Chatterjee, Nachimuthu Senthil Kumar

**Affiliations:** 1 Department of Biotechnology, Mizoram University, Aizawl, Mizoram 796004 India; 2 Human Genetics Unit, Indian Statistical Institute, Kolkata, 700108 India

**Keywords:** Cave, Illumina sequencing, Functional diversity, KEGG pathways, Biospeleology

## Abstract

**Background:**

Bacteria present in cave often survive by modifying their metabolic pathway or other mechanism. Understanding these adopted bacteria and their survival strategy inside the cave is an important aspect of microbial ecology. Present study focuses on the bacterial community and geochemistry in five caves of Mizoram, Northeast India. The objective of this study was to explore the taxonomic composition and presumed functional diversity of cave sediment metagenomes using paired end Illumina sequencing using V3 region of 16S rRNA gene and bioinformatics pipeline.

**Results:**

Actinobacteria, Proteobacteria, Verrucomicrobia and Acidobacteria were the major phyla in all the five cave sediment samples. Among the five caves the highest diversity is found in Lamsialpuk with a Shannon index 12.5 and the lowest in Bukpuk (Shannon index 8.22). In addition, imputed metagenomic approach was used to predict the functional role of microbial community in biogeochemical cycling in the cave environments. Functional module showed high representation of genes involved in Amino Acid Metabolism in (20.9%) and Carbohydrate Metabolism (20.4%) in the KEGG pathways. Genes responsible for carbon degradation, carbon fixation, methane metabolism, nitrification, nitrate reduction and ammonia assimilation were also predicted in the present study.

**Conclusion:**

The cave sediments of the biodiversity hotspot region possessing a oligotrophic environment harbours high phylogenetic diversity dominated by Actinobacteria and Proteobacteria. Among the geochemical factors, ferric oxide was correlated with increased microbial diversity. In-silico analysis detected genes involved in carbon, nitrogen, methane metabolism and complex metabolic pathways responsible for the survival of the bacterial community in nutrient limited cave environments. Present study with Paired end Illumina sequencing along with bioinformatics analysis revealed the essential ecological role of the cave bacterial communities. These results will be useful in documenting the biospeleology of this region and systematic understanding of bacterial communities in natural sediment environments as well.

**Electronic supplementary material:**

The online version of this article (doi:10.1186/s12866-017-1002-x) contains supplementary material, which is available to authorized users.

## Background

Bacteria constitute the major portion of the cave biodiversity and plays a key role in maintaining cave ecosystem [1]. Limited nutrient and energy sources create an oligotrophic environment inside the caves, wherein the primary production is carried out by autotrophic bacteria which inturn supports the growth of several chemo-organotrophic microbes [2]. Bacteria present under this oligotrophic environment often survive by modifying their metabolic pathway or other mechanism [3]. Understanding these adopted bacteria and their survival strategy inside the oligotrophic environment is an important aspect of microbial ecology.

Geomicrobial Investigations in nutrient limited caves are sparse and most of them have been carried out using culture based techniques. Such approach can only detect a minute portion of the total community. Such limitation is solved by the introduction of next generation sequencing (NGS) and expands our knowledge on uncultured microbes [4]. Although the cost of amplicon sequencing (16S rDNA) used for the bacterial community composition studies has rapidly decreased, the functional study using the Shotgun approach or Geochip still remains expensive and thus, is restricted for selected studies [5]. An indirect approach is to compare the uncultured bacterial sequences with closely related and well studied microbes to predict the functional role in the ecosystem. This is also useful to understand the unknown energy source required for metabolism [6, 7]. A computational approach, PICRUSt (phylogenetic investigation of communities by reconstruction of unobserved states) based on the relationship between phylogeny and function was developed to predict functional diversity using 16S rDNA data and a reference database and has been used to study in diverse environments [8].

Cave microorganisms contain a wide range of bacterial groups influenced by the geology, soil or sediment and other factors [9]. Geochemistry parameter often drives the diversity and bacterial community composition inside the caves [10]. Present study focuses on the bacterial community and geochemistry in five unexplored and unknown caves of Mizoram, Northeast India falling under the less- known biodiversity hotspot zone of the eastern Himalayan belt. The objective of this study was to explore the taxonomic composition and to understand how the bacterial communities respond to the cave oligotropic environments. This study was based on the hypothesis that the undisturbed and nutrient- limited cave habitats will host specific bacterial species and the cave geochemical parameters might favour species diversity and richness.

## Methods

### Sample collection and community DNA extraction

**Fig. 1:**
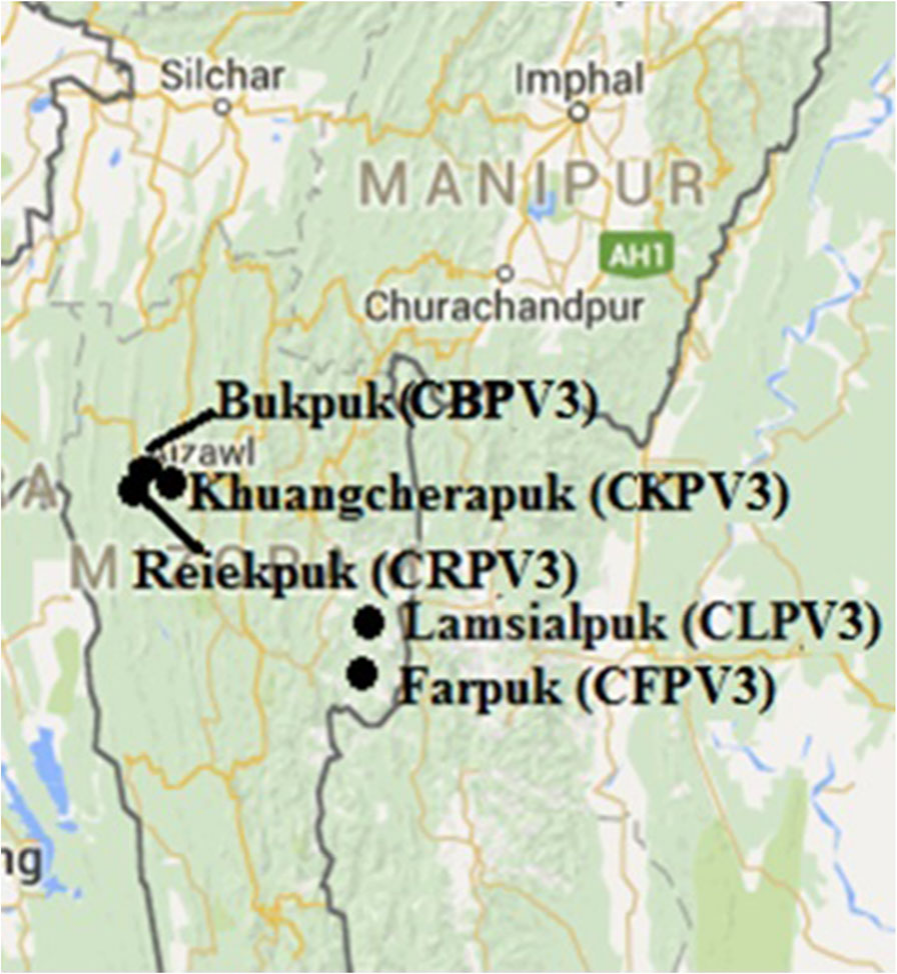
Geographical location of the sampling sites in Mizoram, Northeast India. The figure has been adopted from Google Map and modified

Cave sediment samples were collected from different sites of the caves – Bukpuk (CBP V3), Lamsialpuk (CLP V3) and Reiekpuk (CRP V3) followed by sieved and preserved at 4 °C (Fig. 1). The geochemical and molecular data of the sediment sample Lamsialpuk (CLP V3) and Khuangcherapuk (CKP V3) were collected from our previous study [11, 12]. All sites were not subjected to any human disturbances, except CLPV3 [4]. The elevation, pH and other geochemical parameters of the caves are given in Table 1. The pH of the sediment samples was analysed using pH meter (Eutech, pH 510, USA). Major oxides and trace elements were measured using X-ray Fluorescence (XRF) (Bruker AXS, S4 Pioneer, Germany) at IIT Rookie, India.

**Table 1:** Geochemical parameters of the cave samples

Sample ID	GPS Coordinates	Elevation (MSL)	pH	Na_2_O %	MgO%	Al2O3%	SiO_2_%	P_2_O5%	SO_3_%	K_2_O%	CaO%	Fe2O3%	Cr2O3%	MnO%	NiO%	CuO%	ZnO%	Rb2O%	SrO%	ZrO2%	BaO%	Cl%	V2O5%
CBPV3 (Bukpuk)	N23.69, E93.29	4003	7.2	0.08	1.04	4.59	12.3	7.53	11.9	2.9	8.85	3.21	0.03	0.20	0.03	0.05	0.071	0.03	0.08	0	0.00	0.06	0
CFPV3 (Farpuk)	N23.11, E93.53	4645	7.3	31	0.92	11.8	44.2	0.16	0.16	2.5	0.64	5.15	0.04	0.12	0.02	0.01	0.01	0.01	0.03	0.12	0.07	0	0
CLPV3 (Lamsialpuk)	N23.13, E93.29	4446	7.5	42.4	0.92	13.2	37.9	0.71	0.23	2.7	1.23	6.35	0.03	0.09	0.01	0.01	0.013	0.01	0.03	0.08	0.07	0.06	0.001
CRPV3 (Reiekpuk)	N23.69, E92.60	4312	6.8	0.6	1.67	11.7	39.1	0.16	1.92	2.8	3.23	5.72	0.04	0.09	0.03	0.01	0.013	0.01	0.02	0.07	0.07	0	0
CKPV3 (Khuangcherapuk)	N23.69, E92.61	4900	6.7	0.37	1.04	10.5	33.4	0.84	0.82	2.5	0.92	5.64	0.08	0.05	0.01	0.01	0.012	0.03	0.04	0.07	0.07	0	0

All the samples were collected during March 2014

*MSL* meters above sea level

DNA was extracted from the cave sediment samples using the Fast DNA spin kit (MP Biomedical, Solon, OH, USA) and the V3 hypervariable region of the 16S rRNA gene was amplified using 10 pmol/μl of each forward 341F (5′-CCTACGGGAGGCAGCAG-3′) and reverse 518R (5′-ATTACCGCGGCTGCTGG-3′) primer. PCR Master Mix will contain 2 μL each primers, 0.5 μL of 40 mM dNTP (NEB, USA), 5 μL of 5X Phusion HF reaction buffer (NEB, USA), 0.2 μL of 2 U/μL F-540Special Phusion HS DNA Polymerase (NEB, USA), 5 ng input DNA and water to make up the total volume to 25 μL. The PCR conditions were 98 °C for 30 s followed by 30 cycles of 98 °C for 10 s; 72 °C for 30 s and a final extension at 72 °C for 5 s followed by 4 °C hold.

### Pre-processing and sequence analysis

Paired end Illumina sequencing (2 × 150 bp) was carried out at Scigenome Lab, Cochin, India. Raw sequence data for the two cave sediment samples, Farpuk (CFPV3) and Khuangcherapuk (CKPV3), were derived from our previous study [11, 12]. Raw fastq sequences were processed using the QIIME software package v.1.8.0 [13, 14]. Poor quality (quality score < 25) and smaller reads (read length < 100 bp) were filtered out using the split_libraries command. Pre-processed sequence reads were clustered to operational taxonomic units (OTU’s) using UCLUST method with similarity threshold of 97% [15] and were taxonomically classified using Greengenes database. Relative abundance of the bacterial phyla was calculated using QIIME. Statistical analysis was performed after rarefying the OTU table to 50,000 sequences per sample. Alpha and beta diversity plots were also generated using QIIME. Beta diversity between five bacterial cave communities was measured using unweighted UniFrac approach [16]. Pearson correlations between soil characteristics and bacterial major phylum were estimated using PASW Statistics 18 (SPSS Inc., Chicago, IL, USA). Additionally, we performed imputed metagenomic analysis by the genome prediction software PICRUSt [8]. The input used here was normalized OTU prepared by closed reference based approach. OTU’s were assigned at 97% similarity and were mapped to the Greengenes ver.13.5 database for functional prediction.

### Statistical analysis

Multivariate principal component analysis (PCA) of 20 physicochemical parameters i.e., pH, Na_2_O, MgO, Al_2_O_3_, SiO_2_, P_2_O_5_, SO_3_, K_2_O, CaO, Fe_2_O_3_, Cr_2_O_3_, MnO, NiO, CuO, ZnO, Rb_2_O, SrO, ZrO_2_, BaO and Cl was carried out to determine which environmental variables best explained the observed community patterns using the PAST v3.02 software [17].

## Results

### Geochemical characteristics of the cave sediment samples

The pH of the five cave sediment were recorded in the range of 6.7–7.5. The highest pH was recorded at CLPV3 (7.5) followed by CFPV3 (7.3) and CBPV3 (7.2), whereas the lowest pH was recorded at CKPV3 (6.7). The concentration of the oxides such as Na2O, MgO, Al_2_O_3_, SiO_2_, P_2_O_5_, SO_3_, K_2_O, CaO, CuO, ZnO, Fe_2_O_3_, Cr_2_O_3_, MnO, NiO, CuO, ZnO, Rb_2_O, SrO, ZrO_2_, BaO and Cl varied among the samples (Table 1). Soil samples from both CKPV3 and CRPV3 had similar, but relatively lower pH compared to the other three cave samples. Similarly, CLPV3 and CFPV3 were also geochemically similar with high concentration of Na_2_O (Additional file 1: Figure S1). CBPV3 showed the highest concentration of P_2_O_5_, SO_3_, CaO, MnO, CuO, ZnO and SrO, whereas the lowest concentration of Al_2_O_3_, SiO_2_, Na_2_O and Fe_2_O_3_ compared to other caves. Interestingly, the elevation of CBPV3 was lower than the other four caves under study. A principal component analysis (PCA) of the physicochemical parameters showed that the five caves were separated into four geochemically distinct habitats. The first two principal components explained 88.06% of the total variance. The sample CKPV3 and CRPV3 were found geochemically similar and were grouped together in the 2-dimensional PCA plot. The key influencing parameters for the geochemical diversity were Na_2_O and P_2_O_5_, while Cl and SO_3_ were the other influencing parameters in component 1 and component 2, respectively.

### Analysis of bacterial community composition

**Fig. 2:**
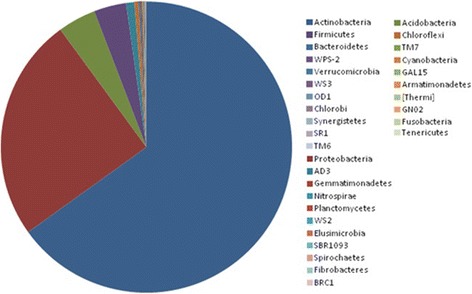
Average bacterial community compositions at the phylum level present in the cave samples

**Fig. 3:**
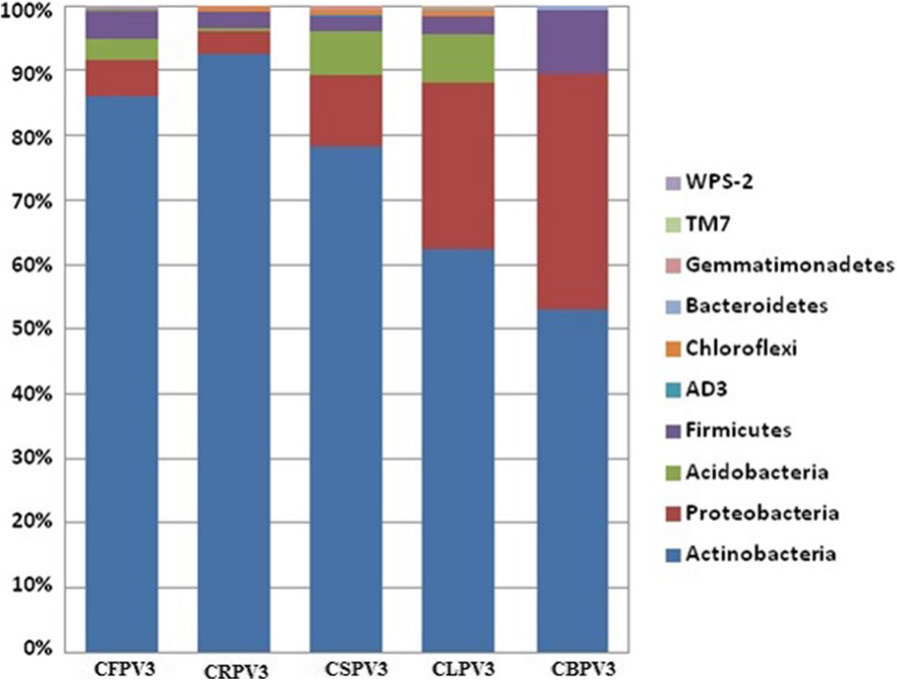
Bacterial community compositions at the phylum level present in the individual cave samples

The high throughput sequencing effort yielded a total of 54,90,239 paired end reads with an average of 9,15,040 paired end reads per sample. After assembly and quality assessment of the reads, a total of 54,88,530 high quality reads were obtained. A total of 48 phyla (AC1, Acidobacteria, Actinobacteria, AD3, Armatimonadetes, Bacteroidetes, BHI80–139, BRC1, Caldithrix, Chlorobi, Chloroflexi, Cyanobacteria, Deferribacteres, Elusimicrobia, FCPU426, Fibrobacteres, Firmicutes, Fusobacteria, GAL15, Gemmatimonadetes, GN02, GN04, MVP-21, NC10, Nitrospirae, NKB19, OD1, OP1, OP11, OP3, OP8, OP9, Planctomycetes, Proteobacteria, SBR1093, SC4, Spirochaetes, SR1, Synergistetes, Tenericutes, Thermi, TM6, TM7, Verrucomicrobia, WPS-2, WS2, WS3 and ZB3) were detected from different cave sediments (Fig. 2). The total bacterial community analysis showed that the phylum Actinobacteria was the most dominant contributing up to 65.1%, followed by Proteobacteria (24.8%), Acidobacteria (4.2%) and Firmicutes (3.6%) and the top ten phyla present in individual cave is shown in Fig. 3.

#### Actinobacteria

In the present study, the identified class under this phylum were Actinobacteria, Acidimicrobiia, Thermoleophilia, Rubrobacteria, MB-A2–108, Coriobacteriia, Nitriliruptoria,

OPB41 and KIST-JJY01. High abundance of dominant family (>0.01%) under Actinobacteria were *Nocardiaceae*, *Streptomycetaceae*, *Micrococcaceae*, *Frankiaceae*, *Gaiellaceae, Pseudonocardiaceae, Streptomycetaceae, EB1017, Mycobacteriaceae, Actinosynnemataceae, Corynebacteriaceae, Rubrobacteraceae, Nocardioidaceae, Micromonosporacea, Geodermatophilaceae, Sporichthyaceae, Actinosynnemataceae, Nakamurellaceae, Pseudonocardiaceae, Cryptosporangiaceae, Kineosporiaceae and Ruaniaceae.* Other dominant genus under Actinomycetes was *Mycobacterium*, *Corynebacterium, Rubrobacter*, *Actinoplanes*, *Saccharothrix* and *Pseudonocardia.*

#### Proteobacteria

Within the Proteobacteria, most phylotypes were classified under the class Alphaproteobacteria and Gammaproteobacteria. Other identified class were Betaproteobacteria, Deltaproteobacteria, Gammaproteobacteria, TA18, Epsilonproteobacteria and Zetaproteobacteria. Abundant genera (≥0.01%) under this phylum were *Rhodoplanes, Kaistobacter*, *Sphingomonas, Bradyrhizobium, Alteromonas, Acidiphilium* and *Halomona.* Under the class Alphaproteobacteria, two families (Hyphomicrobiaceae and Sphingomonadaceae) and three abundant genus (*Rhodoplanes*, *Kaistobacter* and *Sphingomonas*) were identified. Other detected genera, present in low abundance, under this class were *Candidatus entotheonella, Plesiocystis, Desulfococcus*, *Nannocystis*, *Anaeromyxobacter*, *Sorangium*, *Haliangium*, *Geobacter*, *Cystobacter* and *Syntrophus*. The dominant genera under the class Gammaproteobacteria were Alteromonas and Halomonas. Other genera (<0.01%) were *Acinetobacter*, *Alcanivorax*, *Aquicella*, *Cronobacter*, *Dickeya*, *Dokdonella*, *Enhydrobacter*, *Enterobacter*, *Enterovibrio*, *Erwinia*, *Fulvimonas*, *Glaciecola*, *Hafnia*, *Halorhodospira*, *Idiomarina*, *Klebsiella*, *Legionella*, *Luteibacter*, *Luteimonas*, *Marinobacter*, *Marinobacterium*, *Oceanospirillum*, *Providencia*, *Psychrobacter*, *Pseudoxanthomonas*, *Pseudomonas*, *Pseudoalteromonas*, *Rheinheimera*, *Rhodanobacter*, *Salinisphaera*, *Salinivibrio*, *Serpens*, *Serratia*, *Shewanella*, *Stenotrophomonas*, *Steroidobacter* and *Thermomonas*. The class Epsilonproteobacteria was present in low abundance which consisted three families (*Helicobacteraceae*, *Campylobacteraceae* and *Helicobacteraceae*) and two genus (*Arcobacter* and *Sulfurimonas*). However, no genus was identified under the class Zetaproteobacteria and TA18.

#### Acidobacteria

Acidobacteria was the third dominant phyla with eight families and 10 identified genera. Dominant families under this phylum were *Solibacteraceae*, *Koribacteraceae* and *Acidobacteriaceae*. Assigned genera under the family *Acidobacteriaceae* were *Acidobacterium*, *Edaphobacter*, *Terriglobus*, *Acidicapsa* and *Acidopila*.

### Diversity estimates of the cave bacterial community

**Fig. 4:**
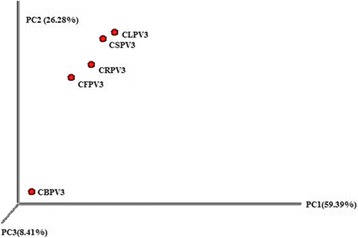
Principal coordinate analysis (PCoA) plot of samples using the unweighted UniFrac distance metric. The variance explained by each principal coordinate axis is shown in parentheses. Datasets were subsample to equal depth prior to the UniFrac distance computation

Based on the Shannon index, a high bacterial diversity was observed in CLPV3 (12.50) and low in CBPV3 (8.22) (Table 2). The principal coordinate analysis plot of the UniFrac distance matrix distinguish CBPV3 from rest of the samples suggesting the presence of different composition of the bacterial communities, whereas other four cave samples had similar community composition (Fig. 4).

**Table 2:** Alpha diversity index of the cave samples

	Observed species	Simpson reciprocal	Shannon	Simpson	PD whole tree
CFPV3	87,179	62.04	9.97	0.001	2914.7
CRPV3	72,638	86.33	10.25	0.001	2357.9
CKPV3	89,805	89.17	11.35	0.001	3020.0
CLPV3	83,136	316.81	12.50	0.004	2873.1
CBPV3	22,004	57.32	8.22	0.003	827.8

All the diversity index is calculated using QIIME

*PD* Phylogenetic Diversity

### Function prediction using PICRUSt

Analysis revealed five functional modules (i.e. metabolism, genetic information processing, environmental information processing, cellular process and organismal systems) where metabolism was the most represented, accounting for about 60% of the entire data set. A deeper analysis of the terms encompassed by the metabolism functional module showed high representation of Amino Acid Metabolism (24%), Carbohydrate Metabolism (23%), Energy Metabolism (10%), Lipid Metabolism (10%), Metabolism of Cofactors and Vitamins (8%), Metabolism of Terpenoids and Polyketides (6%), Nucleotide Metabolism (6%), Metabolism of Other Amino Acids (4%), Enzyme Families (3.0%), Glycan Biosynthesis and Metabolism (3%) and Biosynthesis of Other Secondary Metabolites (3%) (Additional file 1: Figure S2).

With the carbon metabolism, three reactions were involved (carbon degradation, carbon fixation, and methane metabolism). The identified carbon degradation enzymes included genes encoding alpha-amylase, glucoamylase, neopullulanase, and pullulanase (involved in starch degradation); alpha-glucosidase, endoglucanase; beta-glucanase, beta-glucosidase (involved in cellulose degradation); arabinofuranosidase, xylanase, and mannanase (involved in hemicellulose degradation); Chitinase; beta-hexosaminidase; alpha-mannosidase and beta-mannosidase acetyl-glucosaminidase, polygalacturonase (involved in chitin, pectin degradation) and other carbohydrate degradation enzymes. A rare fraction of the predicted metagenomes sequence was classified as 4-hydroxybutyryl-CoA dehydratase. The predicted carbohydrate degrading enzymes were shown in Additional file 1: Table S1.

Gene’s codes for the enzymes methenyltetrahydrofolate cyclohydrolase is also detected in our study. Predicted genes and enzymes show the prevalence of methane cycle in the caves (Additional file 1: Table S2). Analysis also revealed nitrogen cycling genes involved in nitrification, nitrate reduction and ammonia assimilation. Genes codes for the enzyme involved nitronate monooxygenase; nitrile hydratase; nitrate reductase; nitrilase; nitric oxide dioxygenase; nitric oxide reductase; nitric-oxide synthase; nitrite reductase; nitric-oxide reductase; nitrogenase; nitric nitrogen fixation protein; nitroreductase/dihydropteridine reductase; nitrous-oxide reductase; nitroreductase; nitrate reductase; nitrogenase; nitric oxide reductase; nitrogenise (Additional file 1: Table S3).

### Association between bacterial communities with geochemical parameters

A correlation analysis was performed to study the association between the most abundant phyla identified (AD3, Acidobacteria, Actinobacteria, Bacteroidetes, Chloroflexi, Firmicutes, Gemmatimonadetes, Proteobacteria, TM7 and WPS-2) and the geochemical parameters. Analysis revealed that Al_2_O_3_ was positively correlated with Chloroflexi (*r* = 0.627, *p* = 0.060); and MnO was negatively correlated with Acidobacteria (*r* = −0.790, *p* = −0.060). No other relationship between geochemical parameters and the relative abundance of the major phyla was significant different among sampling sites. Within the candidate phyla, MgO was correlated with the relative abundance of the AD3 (*r* = 0.978, *p* = 0.001), TM7 (*r* = 0.974, *p* = −0.001); and WPS-2 (*r* = 0.938, *p* = −0.006) (Additional file 1: Table S4). Furthermore, the content of Fe_2_O_3_ showed highest positive correlation with the Shannon diversity index (*r* = 0.926, *p* = 0.001), followed by Al_2_O_3_, NiO and negative correlation with SO_3_ and MnO (Additional file 1: Table S5).

## Discussion

Speleological studies with NGS approaches are now becoming an important approach for analyzing the concealed microbial diversity in belowground ecosystems [18]. Adaptation of the microorganism in cave ecosystem mostly involves interaction with the minerals, mobilizing inorganic phosphate, oxidizing methane and hydrogen, and deriving energy by hydrolyzing macromolecules derived from other cave microbial communities [19]. High competition for resources in nutrient limited environment helps in natural selection leading to innovation and diversification of bacterial communities [20]. Present study documents the bacterial community composition along with the geochemical analysis of the bacterial community from five different cave sediments in Mizoram, a state of northeast India, situated in Indo-Burma biodiversity hotspot zone.

### Analysis of bacterial community composition

All the cave samples were dominated by the phylum Actinobacteria as seen by our previous study using V4 hypervariable region of 16SrRNA [4].The three most abundant bacterial phyla detected in this study were Actinobacteria a common cave inhabitant has been isolated in rock walls and bioflim of various caves [21]. Isolation of rare and novel Actinobacteria from unexplored environment is an important area of research [22]. Members of the dominant family *Nocardiaceae* have previously been reported in cave ecosystem, are oligotrophic, and can metabolize various substrates such as toluene, herbicides, naphthalene and PCBs [23–27]. The genus *Streptomyces* was the second highest genus falling under the family Streptomycetaceae. Members of this group can metabolize different compounds including alcohols, sugars, amino acids and aromatic compounds and capable of synthesizing clinically useful antibiotics [28].

Proteobacteria was dominated by Alphaproteobacterial species and Gammaproteobacteria. Some species under this subphylum can survive under extreme environment by using ABC (ATP-Binding Cassettes) and TRAP: (Tri-partite ATP-independent periplasmic transporters) mechanism [29]. The genus Rhodoplanes under the subphylum Alphaproteobacteria accounts for 0.15% of the total bacterial community and possesses Photo- and chemo-organ heterotrophic growth [30]. They can also produce hopanoids and carotenoids [31, 32]. Another identified genus under Alphaproteobacteria- *Sphingomona* (0.133%), a group commonly found in nutrient-limited subsurface environments can metabolize a large number of different aromatic compounds [33].

The most abundant genus under Gammaproteobacteria was Alteromonas, a gram negative heterotrophic bacteria capable of degrading aromatic carbon rings introduced through oil spill [34]. Another dominant genus under this subphylum was Halomonas known to resist extreme conditions and also involve in sandstone formations [35]. Among the Betaproteobacteria, the most abundant genera were *Thiobacillus*, *Burkholderia* and *Delftia,* but they were present in less number (<0.002%). *Thiobacillus* can obtain energy by oxidizingo sulfur and ferrous iron compounds [36]. Most of the members under the genus *Burkholderia* were diazotrophs and degrades a variety of xenobiotic compounds [37].

The unique characteristics of the genus *Bdellovibrio* are that they can enter into the periplasmic space of other bacteria and feed on the biopolymers and thereby used as biocontrol purposes [38]. The abundant genus under Acidobacteria was *Candidatus Solibacter,* an aerobic, chemoorganotrophic bacteria having a large number of anion: cation symporters which helps them to survive in nutrient limited condition [39]. Other abundant genus *Candidatus koribacter* was primarily considered as heterotroph [40].

### Metabolic prediction using PICRUSt

The cave environment is a diverse habitat harbouring organisms from all hierarchies starting from prokaryotes to higher eukaryotes [4]. Phylogenetic analysis using 16S SSU-rDNAs data were also applied to assume the metabolic role of the identified bacterial in cave ecosystem by aligning the sequence information to its next nearest culturable representatives [41, 42]. More recently, PICRUSt software package was developed used to infer the potential functional role of bacterial communities in the cave sediment samples using 16S SSU-rDNAs data [8].

Microbial communities are well known key players of biogeochemical cycles and mainly contribute to the global biogeochemical cycling of carbon and nitrogen [29]. Present study detected enzyme 4-hydroxybutyryl-CoA dehydratase involved in the CO_2_-fixation of Archea and fermentation in bacteria which supports the hypothesis that autotrophic archaea contribute to carbon assimilation in cave and other environments [43–45]. Analysis also detected methenyltetrahydrofolate cyclohydrolase which is involved in reverse methanogenesis prevalent in anaerobic methanotrophic archaea [46, 47]. The presence of genes encoding proteins for the phosphate recycling mechanism, such as phosphonate transpoters (PhnB, PhnG, PhnH, PhnI, PhnJ, and PhnM) in the cave samples suggest that they form carbonphosphorus lyase complex which is involved in methane production from methyl phosphonate [48].

Role of bacteria in nitrogen cycle have been well studied in soil and aquatic habitats, but information on cave sediment is limited. Some reports are available where microbes can accrue energy as well as nutrients in oligotrophic environments through nitrogen cycling processes. Most of the genes involved in nitrogen cycle were detected in the present study. Presence of the genes codes for hydroxylamine oxidase indicates the presence of a key ammonia oxidizing bacteria (AOB) [49]. Presence of AOB and sulfur-oxidizing bacteria were also reported in chemolithotrophic Cave [48] and thus lithochemotrophy might be a survival strategy of the bacterial communities present in the cave sediments. Identified genus, *Nitrospira* and *Nitrosospira* were reported to perform autotrophic nitrification which is an indication of CO_2_-fixation-coupled ammonia oxidation process in the studied cave ecosystems [50].

### Association between bacterial communities with geochemical parameters

Bacterial community structure is greatly influenced by the mineral substrates present in an environment [51]. Present study observed the positive relationship between Fe_2_O_3_, Al_2_O_3_ and NiO with the Shannon diversity index. Fe (II) is produced on the subsurface under anoxic conditions by dissimilatory iron (III) reducing bacteria (DIRB) coupled with biotic/abiotic weathering of minerals. Reduced metals inside the cave serve as a source of electron donor for bacterial growth [52, 53]. Only certain organisms can survive in the presence of oligotrophic forces and a high-metal environment, and the natural selection favours adaptations in microbial communities to sustain in these environments.

## Conclusion

Present study used Illumina sequencing to examine the taxonomical diversity of bacterial communities present in cave sediment samples, which were collected from Mizoram, an Indo-Burma Biodiversity Hotspot. These oligotrophic cave harbours a high phylogenetic diversity, including organisms from all hierarchies as well as a higher proportion of unclassified sequences indicating the possibility of novel species. The cave sediments were dominated by Actinobacteria and Proteobacteria. Fe_2_O_3_ content was correlated with increased microbial diversity in these cave environments. Bioinformatics analysis detected genes involved in various metabolic pathways which are essential for the survival of the community in nutrient limited cave environments. Further research by cultivating the uncultured communities or whole genome sequencing is needed to illustrate the actual survival strategies in the cave environments.

## Additional file

Additional file 1: Table S1. List of the genes codes for enzymes involved in carbohydrate degradation identified using PICRUSt. **Table S2.** List of the homologs of methanogenesis-associated genes that were identified from the five cave sediments using PICRUSt. **Table S3.** List of the genes coding for enzymes involved in nitrogen cycle identified using PICRUSt. **Table S4.** Pearson correlation (PC) between physiochemical factors with the dominant bacterial phyla. **Table S5.** Pearson correlation (PC) between physiochemical factors with the bacterial diversity. **Figure S1.** Bioplot generated for the Principal Component Analysis (PCA) of 20 geochemical variables. Cave samples are shown as colored symbols and physicochemical variables are represented by green lines. **Figure S2.** Relative abundance of the functional genes present in the cave samples. (DOCX 122 kb)

## Abbreviations

ABC: ATP-Binding Cassettes
AOB: ammonia oxidizing bacteria
HMP: human microbiome project
NGS: next generation sequencing
PCA: principal component analysis
PICRUSt: phylogenetic investigation of communities by reconstruction of unobserved states
TRAP: tri-partite ATP-independent periplasmic transporters
