# Supplementary material for: Dominant bacterial phyla in caves and their predicted functional roles in C and N cycle
Source: BMC Microbiol. 2017 Apr 11;17:90. doi: 10.1186/s12866-017-1002-x (PMC5387202; doi:10.1186/s12866-017-1002-x)
Supplement: Additional file 1: Table S1. — List of the genes codes for enzymes involved in carbohydrate degradation identified using PICRUSt. Table S2. List of the homologs of methanogenesis-associated genes that were identified from the five cave sediments using PICRUSt. Table S3. List of the genes coding for enzymes involved in nitrogen cycle identified using PICRUSt. Table S4. Pearson correlation (PC) between physiochemical factors with the dominant bacterial phyla. Table S5. Pearson correlation (PC) between physiochemical factors with the bacterial diversity. Figure S1. Bioplot generated for the Principal Component Analysis (PCA) of 20 geochemical variables. Cave samples are shown as colored symbols and physicochemical variables are represented by green lines. Figure S2. Relative abundance of the functional genes present in the cave samples. (DOCX 122 kb) [file 12866_2017_1002_MOESM1_ESM.docx]

**Table S1.** **List of the genes codes for enzymes involved in carbohydrate degradation identified using PICRUST.**

| **OTU ID** | **CFPV3** | **CRPV3** | **CKPV3** | **CLPV3** | **CBPV3** | **KEGG_Description** |
| --- | --- | --- | --- | --- | --- | --- |
| K01176 | 27391 | 18576 | 20919 | 12211 | 5010 | alpha-amylase [EC:3.2.1.1] |
| K07405 | 236 | 177 | 424 | 275 | 36 | alpha-amylase [EC:3.2.1.1] |
| K01178 | 15132 | 4170 | 20708 | 16034 | 329 | glucoamylase [EC:3.2.1.3] |
| K01179 | 150806 | 123986 | 108567 | 78023 | 33916 | endoglucanase [EC:3.2.1.4] |
| K01190 | 109617 | 53776 | 98347 | 58180 | 27861 | beta-galactosidase [EC:3.2.1.23] |
| K12308 | 104500 | 87697 | 91238 | 68506 | 12062 | beta-galactosidase [EC:3.2.1.23] |
| K05350 | 224167 | 177804 | 157940 | 97063 | 31122 | beta-glucosidase [EC:3.2.1.21] |
| K05349 | 193177 | 146916 | 161768 | 114774 | 40231 | beta-glucosidase [EC:3.2.1.21] |
| K01188 | 95216 | 80758 | 76506 | 48365 | 23967 | beta-glucosidase [EC:3.2.1.21] |
| K01176 | 27391 | 18576 | 20919 | 12211 | 5010 | alpha-amylase [EC:3.2.1.1] |
| K07405 | 236 | 177 | 424 | 275 | 36 | alpha-amylase [EC:3.2.1.1] |
| K07407 | 138423 | 113472 | 125872 | 79099 | 15619 | alpha-galactosidase [EC:3.2.1.22] |
| K07406 | 5063 | 5331 | 6700 | 7014 | 151 | alpha-galactosidase [EC:3.2.1.22] |
| K01187 | 313018 | 217131 | 230125 | 150604 | 54696 | alpha-glucosidase [EC:3.2.1.20] |
| K01183 | 121687 | 115347 | 96013 | 64813 | 21106 | chitinase [EC:3.2.1.14] |
| K12373 | 100409 | 85729 | 84596 | 56538 | 17836 | beta-hexosaminidase [EC:3.2.1.52] |
| K01191 | 64145 | 30009 | 47197 | 28082 | 8375 | alpha-mannosidase [EC:3.2.1.24] |
| K01192 | 49129 | 27158 | 43721 | 27791 | 7200 | beta-mannosidase [EC:3.2.1.25] |
| K01180 | 1942 | 839 | 643 | 235 | 1095 | endo-1,3(4)-beta-glucanase [EC:3.2.1.6] |
| K01184 | 11671 | 1911 | 16848 | 15166 | 285 | polygalacturonase [EC:3.2.1.15] |
| K01181 | 95826 | 91753 | 87453 | 60145 | 19350 | endo-1,4-beta-xylanase [EC:3.2.1.8] |
| K01209 | 92946 | 40905 | 99482 | 73730 | 20782 | alpha-N-arabinofuranosidase [EC:3.2.1.55] |
| K01180 | 1942 | 839 | 643 | 235 | 1095 | endo-1,3(4)-beta-glucanase [EC:3.2.1.6] |
| K01234 | 2524 | 1258 | 922 | 374 | 1125 | neopullulanase [EC:3.2.1.135] |
| K01200 | 2681 | 1405 | 999 | 871 | 1178 | pullulanase [EC:3.2.1.41] |

**Table S2. List of the homologs of methanogenesis-associated genes that were identified from the five cave sediments using PICRUST.**

| **OTU ID** | **CFPV3** | **CRPV3** | **CKPV3** | **CLPV3** | **CBPV3** | **KEGG_Description** |
| --- | --- | --- | --- | --- | --- | --- |
| **K00200** | 535 | 283 | 1446 | 1060 | 55 | formylmethanofuran dehydrogenase subunit A [EC:1.2.99.5] |
| **K00201** | 532 | 279 | 1442 | 1058 | 54 | formylmethanofuran dehydrogenase subunit B [EC:1.2.99.5] |
| **K00202** | 532 | 279 | 1442 | 1058 | 54 | formylmethanofuran dehydrogenase subunit C [EC:1.2.99.5] |
| **K11261** | 13727 | 2252 | 18636 | 14160 | 132 | formylmethanofuran dehydrogenase subunit E [EC:1.2.99.5] |
| **K00672** | 237 | 168 | 693 | 873 | 50 | Formylmethanofuran - tetrahydromethanopterin N-formyltransferase [EC:2.3.1.101] |
| **K01433** | 144630 | 163314 | 95958 | 61705 | 38186 | formyltetrahydrofolate deformylase [EC:3.5.1.10] |
| **K01499** | 315 | 256 | 876 | 907 | 51 | methenyltetrahydromethanopterin cyclohydrolase [EC:3.5.4.27] |
| **K00320** | 235910 | 197179 | 146818 | 61800 | 36883 | coenzyme F420-dependent N5,N10-methenyltetrahydro methanopterin reductase [EC:1.5.99.11] |
| **K03388** | 1717 | 439 | 1239 | 351 | 144 | heterodisulfide reductase subunit A [EC:1.8.98.1] |

**Table S3. List of the genes coding for enzymes involved in nitrogen cycle identified using PICRUST.**

| **OTU ID** | **CFPV3** | **CRPV3** | **CKPV3** | **CLPV3** | **CBPV3** | **Average** | **KEGG_Description** |
| --- | --- | --- | --- | --- | --- | --- | --- |
| **K00459** | 234608 | 181071 | 171865 | 98890 | 44603 | 145164.3 | nitronate monooxygenase [EC:1.13.12.16] |
| **K04751** | 148620 | 153605 | 119582 | 79297 | 31707 | 112312.2 | nitrogen regulatory protein P-II |
| **K00362** | 186707 | 187798 | 122042 | 66941 | 42569 | 106609 | nitrite reductase (NAD(P)H) large subunit |
| **K00363** | 158467 | 139566 | 112051 | 60392 | 37056 | 93796.17 | nitrite reductase (NAD(P)H) small subunit |
| **K04488** | 158002 | 114409 | 103742 | 65094 | 19771 | 86914.83 | nitrogen fixation protein NifU and related proteins |
| **K01721** | 109460 | 180569 | 59540 | 40267 | 9425 | 72093 | nitrile hydratase [EC:4.2.1.84] |
| **K01501** | 96026 | 84391 | 49338 | 20949 | 9501 | 44251 | nitrilase [EC:3.5.5.1] |
| **K05916** | 80284 | 56745 | 56551 | 30192 | 11872 | 41549.67 | nitric oxide dioxygenase [EC:1.14.12.17] |
| **K00372** | 58741 | 73302 | 39085 | 30134 | 23134 | 40560.67 | nitrate reductase catalytic subunit [EC:1.7.99.4] |
| **K00491** | 52136 | 65495 | 27735 | 9571 | 4284 | 26694.33 | nitric-oxide synthase, bacterial [EC:1.14.13.39] |
| **K00373** | 47649 | 33337 | 31306 | 20412 | 6985 | 25441.17 | nitrate reductase 1, delta subunit [EC:1.7.99.4] |
| **K00370** | 44072 | 34009 | 26246 | 19227 | 11699 | 24024.67 | nitrate reductase 1, alpha subunit [EC:1.7.99.4] |
| **K04561** | 9594 | 2498 | 15692 | 11101 | 6918 | 21192.67 | nitric oxide reductase, cytochrome b-containing subunit I |
| **K00371** | 41407 | 28771 | 25127 | 17640 | 6345 | 21156.83 | nitrate reductase 1, beta subunit [EC:1.7.99.4] |
| **K00374** | 40555 | 28424 | 24419 | 17059 | 6319 | 20598 | nitrate reductase 1, gamma subunit [EC:1.7.99.4] |
| **K00368** | 2577 | 5076 | 6234 | 7950 | 616 | 16838.17 | nitrite reductase (NO-forming) [EC:1.7.2.1] |
| **K02164** | 7387 | 11805 | 13312 | 9168 | 8882 | 16108.5 | nitric-oxide reductase NorE protein [EC:1.7.99.7] |
| **K04752** | 6223 | 3546 | 10948 | 16443 | 11953 | 10241.67 | nitrogen regulatory protein P-II 2 |
| **K02586** | 6826 | 7199 | 10569 | 13251 | 12152 | 9190.333 | nitrogenase molybdenum-iron protein alpha chain |
| **K02591** | 6813 | 7198 | 10560 | 13179 | 12170 | 9174.167 | nitrogenase molybdenum-iron protein beta chain [EC:1.18.6.1] |
| **K02588** | 6667 | 7102 | 10213 | 12193 | 12170 | 8682.333 | nitrogenase iron protein NifH [EC:1.18.6.1] |
| **K04748** | 1774 | 2178 | 8745 | 9454 | 712 | 7596 | nitric oxide reductase NorQ protein |
| **K02596** | 5149 | 6555 | 7535 | 11161 | 5653 | 6762.333 | nitrogen fixation protein NifX |
| **K02587** | 5098 | 6495 | 7236 | 10825 | 5659 | 6634 | nitrogenase molybdenum-cofactor synthesis protein NifE |
| **K02585** | 5357 | 6440 | 7586 | 10047 | 5703 | 6375.333 | nitrogen fixation protein NifB |
| **K02592** | 4259 | 6127 | 6382 | 9626 | 5648 | 5775 | nitrogenase molybdenum-iron protein NifN |
| **K02595** | 4138 | 6109 | 6228 | 9585 | 5643 | 5717.167 | nitrogen fixation protein NifW |
| **K02597** | 1979 | 982 | 6781 | 10361 | 333 | 4090.333 | nitrogen fixation protein NifZ |
| **K02593** | 1203 | 470 | 4719 | 7648 | 144 | 2581.167 | nitrogen fixation protein NifT |
| **K00360** | 1336 | 6314 | 3699 | 2612 | 61 | 2359.833 | nitrate reductase (NADH) [EC:1.7.1.1] |
| **K10679** | 3126 | 1955 | 1260 | 1101 | 1432 | 1560.833 | nitroreductase / dihydropteridine reductase [EC:1.-.-.-1.5.1.34] |
| **K07218** | 1232 | 2243 | 1497 | 1943 | 112 | 1241.333 | nitrous oxidase accessory protein |
| **K00376** | 1225 | 2240 | 1493 | 1908 | 90 | 1228.833 | nitrous-oxide reductase [EC:1.7.99.6] |
| **K10678** | 421 | 299 | 677 | 1183 | 284 | 713.3333 | nitroreductase [EC:1.-.-.-] |
| **K02305** | 316 | 339 | 865 | 1441 | 193 | 680.8333 | nitric-oxide reductase, cytochrome c-containing subunit II |
| **K02448** | 230 | 319 | 755 | 1426 | 192 | 642 | nitric-oxide reductase NorD protein [EC:1.7.99.7] |
| **K02598** | 385 | 630 | 588 | 976 | 13 | 436.1667 | nitrite transporter NirC |
| **K00369** | 28 | 37 | 87 | 338 | 14 | 394.6667 | nitrate reductase [EC:1.7.99.4] |
| **K02590** | 578 | 144 | 207 | 60 | 13 | 250.6667 | nitrogen regulatory protein PII 2 |
| **K02589** | 578 | 144 | 206 | 59 | 13 | 250.1667 | nitrogen regulatory protein PII 1 |
| **K00531** | 193 | 72 | 577 | 431 | 5 | 214.8333 | nitrogenase [EC:1.18.6.1] |
| **K12265** | 55 | 23 | 15 | 10 | 29 | 22.33333 | nitric oxide reductase FlRd-NAD(+) reductase [EC:1.18.1.-] |
| **K04747** | 2 | 11 | 14 | 15 | 0 | 7 | nitric oxide reductase NorF protein |
| **K08345** | 20 | 10 | 2 | 2 | 0 | 5.666667 | nitrate reductase 2, alpha subunit [EC:1.7.99.4] |
| **K08346** | 20 | 10 | 2 | 2 | 0 | 5.666667 | nitrate reductase 2, beta subunit [EC:1.7.99.4] |
| **K08361** | 20 | 10 | 2 | 2 | 0 | 5.666667 | nitrate reductase 2, delta subunit [EC:1.7.99.4] |
| **K08347** | 20 | 10 | 2 | 2 | 0 | 5.666667 | nitrate reductase 2, gamma subunit [EC:1.7.99.4] |
| **K10851** | 2 | 2 | 1 | 5 | 5 | 2.833333 | nitrogen regulatory protein A |
| **K00536** | 1 | 0 | 6 | 6 | 2 | 2.666667 | nitrogenase (flavodoxin) [EC:1.19.6.1] |

**Table S4: Pearson correlation (PC) between physiochemical factors with the dominant bacterial phyla.**

|  | **pH** | **Na2O** | **MgO** | **Al2O3** | **SiO2** | **P2O5** | **SO3** | **K2O** | **CaO** | **Fe2O3** | **Cr2O3** | **MnO** | **NiO** | **CuO** | **ZnO** | **Rb2O** | **SrO** | **ZrO2** | **BaO** | **Cl** |
| --- | --- | --- | --- | --- | --- | --- | --- | --- | --- | --- | --- | --- | --- | --- | --- | --- | --- | --- | --- | --- |
| **AD3** | -0.38 | -0.39 | 0.98^**^ | 0.26 | 0.29 | -0.28 | -0.08 | 0.26 | 0.09 | 0.09 | -0.11 | -0.14 | 0.20 | -0.22 | -0.20 | -0.42 | -0.36 | 0.09 | -0.04 | -0.44 |
| **Acidobacteria** | -0.77 | -0.36 | 0.38 | 0.26 | 0.10 | -0.45 | -0.44 | -0.38 | -0.4 | 0.65 | 0.36 | -0.79 | 0.16 | -0.47 | -0.47 | 0.43 | 0.14 | -0.07 | 0.58 | -0.12 |
| **Actinobacteria** | 0.32 | 0.018 | -0.69 | -0.58 | -0.68 | 0.704 | 0.551 | 0.12 | 0.422 | -0.43 | 0.14 | 0.43 | -0.07 | 0.638 | 0.642 | 0.46 | 0.33 | -0.55 | -0.34 | 0.58 |
| **Bacteroidetes** | 0.287 | 0.45 | -0.33 | 0.25 | 0.48 | -0.33 | -0.34 | -0.24 | -0.39 | 0.004 | -0.13 | 0.05 | -0.21 | -0.3 | -0.32 | -0.33 | -0.12 | 0.63 | 0.12 | -0.35 |
| **Chloroflexi** | -0.74 | -0.64 | 0.804 | -0.06 | -0.11 | -0.11 | 0.015 | -0.11 | 0.12 | 0.23 | -0 | -0.29 | 0.54 | -0.07 | -0.08 | 0.19 | 0.14 | -0.33 | 0.26 | -0.12 |
| **Firmicutes** | 0.68 | 0.72 | -0.62 | 0.31 | 0.43 | -0.19 | -0.27 | 0.04 | -0.33 | -0.08 | -0.07 | 0.143 | -0.56 | -0.21 | -0.2 | -0.42 | -0.29 | 0.63 | -0.13 | -0.14 |
| **Gemmatimonadetes** | -0.34 | 0.004 | -0.47 | 0.01 | -0.19 | -0.13 | -0.28 | -0.48 | -0.39 | 0.48 | 0.46 | -0.58 | -0.08 | -0.2 | -0.21 | 0.73 | 0.38 | -0.19 | 0.49 | 0.30 |
| **Proteobacteria** | -0.57 | -0.29 | 0.76 | 0.32 | 0.41 | -0.53 | -0.38 | -0.25 | -0.27 | 0.38 | -0.12 | -0.4 | 0.35 | -0.46 | -0.48 | -0.18 | -0.08 | 0.25 | 0.4 | -0.48 |
| **TM7** | -0.39 | -0.40 | 0.97^**^ | 0.25 | 0.28 | -0.28 | -0.09 | 0.23 | 0.085 | 0.11 | -0.13 | -0.15 | 0.23 | -0.22 | -0.21 | -0.39 | -0.33 | 0.08 | -0.01 | -0.43 |
| **WPS-2** | -0.48 | -0.41 | 0.94^**^ | 0.24 | 0.23 | -0.31 | -0.14 | 0.09 | 0.025 | 0.24 | -0.13 | -0.26 | 0.32 | -0.26 | -0.25 | -0.25 | -0.20 | 0.01 | 0.14 | -0.32 |

** - indicates P value <0.001 and is highly significant.

**Table S5: Pearson correlation (PC) between physiochemical factors with the bacterial diversity.**

|  | **pH** | **Na_2_O** | **MgO** | **Al_2_O_3_** | **SiO_2_** | **P_2_O_5_** | **SO_3_** | **K_2_O** | **CaO** | **Fe_2_O_3_** | **Cr_2_O_3_** | **MnO** | **NiO** | **CuO** | **ZnO** | **Rb_2_O** | **SrO** | **ZrO_2_** | **BaO** | **Cl** |
| --- | --- | --- | --- | --- | --- | --- | --- | --- | --- | --- | --- | --- | --- | --- | --- | --- | --- | --- | --- | --- |
| Shannon | 0.09 | 0.55 | -0.16 | 0.83 | 0.63 | -0.73 | -0.81 | -0.48 | -0.79 | 0.93^**^ | 0.28 | -0.82 | -0.84 | -0.78 | -0.76 | -0.38 | -0.67 | 0.55 | 0.78 | -0.06 |

** - indicates P value <0.001 and is highly significant.


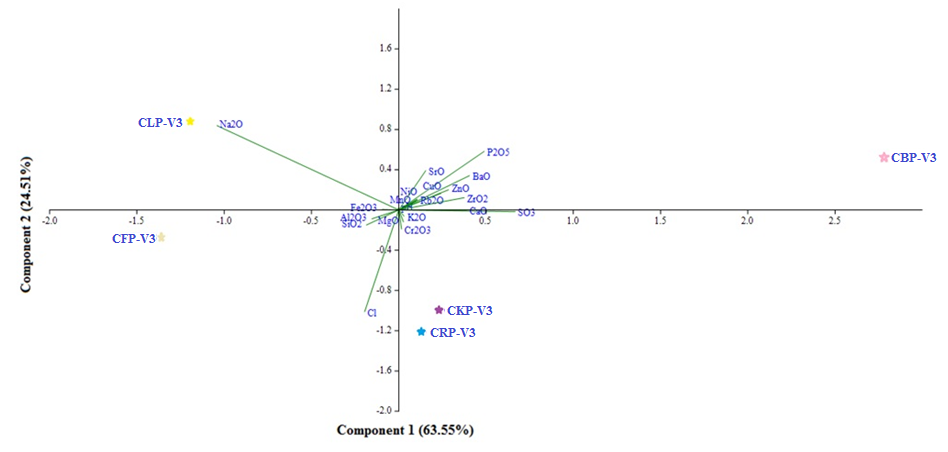


**Supplementary Figure 1:** Bioplot generated for the Principal Component Analysis (PCA) of twenty geochemical variables. Cave samples are shown as colored symbols and physicochemical variables are represented by green lines.

**Supplementary Figure 2:** Relative abundance of the functional genes present in the cave samples.
